# Quantitative confocal imaging method for analyzing cellulose dynamics during cell wall regeneration in Arabidopsis mesophyll protoplasts

**DOI:** 10.1002/pld3.21

**Published:** 2017-12-27

**Authors:** Hiroaki Kuki, Takumi Higaki, Ryusuke Yokoyama, Takeshi Kuroha, Naoki Shinohara, Seiichiro Hasezawa, Kazuhiko Nishitani

**Affiliations:** ^1^ Graduate School of Life Sciences Tohoku University Sendai Japan; ^2^ International Research Organization for Advanced Science and Technology Kumamoto University Kumamoto Japan; ^3^ Department of Integrated Biosciences Graduate School of Frontier Sciences The University of Tokyo Kashiwanoha Kashiwa Chiba Japan

**Keywords:** *Arabidopsis thaliana*, cell wall, cellulose microfibrils, imaging, protoplast, quantitative image analysis, regeneration

## Abstract

The network structure of cellulose fibrils provides mechanical properties to the primary cell wall, thereby determining the shapes and growth patterns of plant cells. Despite intensive studies, the construction process of the network structure *in muro* remains largely unknown, mainly due to the lack of a robust, straightforward technique to evaluate network configuration. Here, we developed a quantitative confocal imaging method for general use in the study of cell wall dynamics in protoplasts derived from Arabidopsis leaf mesophyll cells. Confocal imaging of regenerating cell walls in protoplasts stained with Calcofluor allowed us to visualize the cellulose network, comprising strings of bundled cellulosic fibrils. Using image analysis techniques, we measured several metrics including total length, which is a measure of the spread of the cellulose network. The total length increased during cell wall regeneration. In a proof‐of‐concept experiment using microtubule‐modifying agents, oryzalin, an inhibitor of microtubule polymerization, inhibited the increase in total length and caused abnormal orientation of the network, as shown by the decrease in the average angle of the cellulose with respect to the cell long axis. Taxol, a microtubule stabilizer, stimulated the bundling of cellulose fibrils, as shown by the increase in skewness in the fluorescence intensity distribution of Calcofluor, and inhibited the increase in total length. These results demonstrate the validity of this method for quantitative imaging of the cellulose network, providing an opportunity to gain insight into the dynamic aspects of cell wall regeneration.

## 1 INTRODUCTION

The primary cell wall in land plants is a supermolecular network primarily composed of cellulose and matrix polymers (Cosgrove, 2005; Somerville et al., 2004; Yokoyama, Shinohara, Asaoka, Narukawa, & Nishitani, 2015). Cellulose is present in the form of crystalline microfibrils, each composed of a few dozen β‐1,4‐glucans. This network provides the load‐bearing framework of the primary cell wall, thereby playing a pivotal role in determining cell shape and, hence, plant organ structure. A single cellulose microfibril is synthesized by a single cellulose synthase complex (CSC) comprising six subunits, each containing multiple cellulose synthases (Kimura et al., 1999; McFarlane, Doring, & Persson, 2014).

The orientation of newly deposited cellulose microfibrils, which is guided by cortical microtubules (Paredez, Somerville, & Ehrhardt, 2006), is thought to be responsible for determining the direction of cell expansion (Baskin, 2005). Cellulose fibrils become reoriented during cell wall expansion in certain cells, such as *Arabidopsis thaliana* root cells, indicating that cellulose fibrils are dynamic, even after they are deposited in the cell wall (Anderson, Carroll, Akhmetova, & Somerville, 2010). Several approaches have been utilized to gain insight into the dynamic aspects of cellulose network structure. These approaches include optical observation of the inner surface of the cell wall via atomic force microscopy or field emission scanning electron microscopy (Himmelspach, Williamson, & Wasteneys, 2003; Sugimoto, Himmelspach, Williamson, & Wasteneys, 2003; Sugimoto, Williamson, & Wasteneys, 2000; Zhang, Vavylonis, Durachko, & Cosgrove, 2017; Zhang, Zheng, & Cosgrove, 2016) and fluorescence microscopic observation of cellulose stained with cellulose‐specific dyes (Anderson et al., 2010). Despite the extensive optical studies, it is still a challenge to quantitatively characterize the deposition patterns of cellulose microfibrils on the plasma membrane during construction and remodeling of the primary cell wall.

In this study, we designed a new imaging technique to quantitatively evaluate the cellulose network configuration during cell wall regeneration in protoplasts derived from rosette leaves of Arabidopsis. For this process, we coupled a conventional technique for cellulose staining (Nagata and Takebe 1970; Hayashi, Polonenko, Camirand, & Maclachlan, 1986; Shea, Gibeaut, & Carpita, 1989; Fisher and Cyr 1998) with an image analysis technique used to quantitatively evaluate the configuration of the cytoskeleton (Higaki, Kutsuna, Sano, Kondo, & Hasezawa, 2010; Kimata et al., 2016; Ueda et al., 2010; Yoneda et al., 2007). By combining our image analysis approach with a high‐yielding cell wall regeneration procedure, we successfully characterized the network patterns of nascent cellulose in protoplasts stained with Calcofluor White M2R (Calcofluor) in a quantitative manner. We also examined the effects of oryzalin and taxol, a destabilizer and stabilizer of microtubules, respectively, on the cellulose network during cell wall regeneration as a proof‐of‐concept.

## 2 EXPERIMENTAL PROCEDURES

### 2.1 Plant materials

*Arabidopsis thaliana* (L.) Heynh. ecotype Col‐0 was used as the wild type. A transgenic Arabidopsis line expressing *UBQ10::GFP‐TUB6* in the Col‐0 background (Nakamura, Naoi, Shoji, & Hashimoto, 2004) was used to visualize cortical microtubules. In all experiments, seeds from the wild type and transgenic line were sown on Rockwool blocks (Grodan, Rockwool B.V.) moistened with MGRL medium (Tsukaya, Ohshima, Naito, Chino, & Komeda, 1991) and grown under continuous light (60–70 μmol m^−2^ s^−1^) at 22°C in a growth chamber.

### 2.2 Preparation and incubation of protoplasts

Protoplasts were isolated according to Yoo, Cho, and Sheen (2007) with some modifications. Fully expanded rosette leaves of 20‐day‐old Arabidopsis plants were detached and sterilized by immersion in 70% ethanol for 30 s and 4% (v/v) sodium hypochlorite solution for 30 s, followed by two washes in 0.45 M mannitol solution. Sterilized leaves were cut into strips and immersed in 15 mL of enzyme solution (1% cellulase R10 and 0.4% macerozyme R10 [Yakult Pharmaceutical Ind. Co. Japan], 0.45 M mannitol, 20 mM KCl, 10 mL of CaCl_2_, and 20 mM 2‐morpholinoethanesulfonic acid [MES]; pH 5.7) in a Petri dish (9 cm diameter). The immersed specimens were infiltrated in the solution under reduced pressure for 10 min, followed by incubation for 5 hr at room temperature and atmospheric pressure. After the incubation, protoplasts were released from the tissue specimen by gentle shaking, and an equal volume of W5 solution (2 mM MES, 154 mM NaCl, 125 mM CaCl_2_, 5 mM KCl; pH 5.7) was added to suspend the protoplasts. The protoplasts were filtered through 100‐μm and 50‐μm nylon meshes to remove large tissue debris, collected by centrifugation at 100 *g* for 2 min, resuspended in W5 solution, and collected again by centrifugation. The protoplasts were resuspended in regeneration medium (Gamborg's B‐5 basal medium with minimal organics [SIGMA], 0.4 M trehalose, 0.05 M glucose, and 1 μM 3‐naphthalene acetic acid; pH 5.7) and incubated in continuous light (60–70 μmol m^−2^ s^−1^) at 22°C to regenerate cell walls. For the inhibitor experiments, 10 μM oryzalin, 10 μM taxol, or 0.3 μM isoxaben dissolved in 0.01% DMSO was added to the regeneration medium; 0.01% DMSO was used as a control.

### 2.3 Protoplast staining and image acquisition for cellulose fibrils

Protoplasts at various stages of cell wall regeneration were stained with 0.001% Calcofluor (SIGMA) for 5 min or with 0.03% Direct Red 23 (the same dye as S4B) (SIGMA) for 30 min. For callose staining, protoplasts were transferred to 0.05% aniline blue solution (0.05% aniline blue, 0.1 M K_2_HPO_4_, 0.4 M trehalose; pH 8.7). Images were acquired under a confocal laser scanning microscope (FV‐1000‐D; Olympus) using laser beam lines of 405 nm for Calcofluor or aniline blue, 473 nm for GFP‐TUB6, or 559 nm for S4B. Serial optical sectional images from the top to the middle of the protoplast were acquired at 0.5‐μm intervals.

### 2.4 Image processing

MIP images were obtained from the serial optical sectional images and skeletonized using ImageJ plug‐ins: LpxLineExtract, which is invoked by Lpx_Filter2d plug‐ins (filter = lineFilters, linemode = lineExtract) in the LPixel ImageJ plugins package (available for free at https://lpixel.net/services/research/lpixel-imagej-plugins/). The parameters were as follows: giwsIter = 8, mdnmsLen = 7, pickup = above, shaveLen = 5, and delLen = 5. The skeletonized images were masked with manual segmentation of the target protoplast regions. The masked skeletonized images were used to measure metrics that quantitatively evaluate the configurations of the cellulose network as described below.

To quantitatively evaluate the spread of the cellulose network, the number of pixels constituting the cellulose microfibrils (*N*_cellulose_) was measured and converted to millimeters (mm) of total length. The mean fluorescent intensity of the skeletonized cellulose pixels ($\overset{¯}{i}$) was measured to evaluate cellulose levels.

To estimate the bundling configuration of cellulose fibrils, we measured skewness (S) of intensity distribution, which is the degree of asymmetry of intensity distribution, as defined by

$$S=\frac{1}{N_{\mathrm{cellulose}}}\sum_{i=1}^{N_{\mathrm{cellulose}}}{\left(\frac{i_{n}-\overset{¯}{i}}{\sigma }\right)}^{3}$$

$$\sigma =\frac{1}{N_{\mathrm{cellulose}}}\sum_{i=1}^{N_{\mathrm{cellulose}}}{\left(i_{n}-\overset{¯}{i}\right)}^{2}$$

where S increases as the shift and skews the intensity distribution to the left on the horizontal axis, which indicates the fluorescence intensities (Higaki et al., 2010).

To estimate cellulose orientation, the angular difference between the mean angle of cellulose microfibrils and the long axis (∆θ) of the cell was measured to represent the average angle, basically as described (Kimata et al., 2016). ∆θ was defined as

$$\Delta \theta =\left\{\left\{\begin{array}{cc} |\theta_{\mathrm{protoplast}}-\theta_{\mathrm{cellulose}}| & if|\theta_{\mathrm{protoplast}}-\theta_{\mathrm{cellulose}}|\leq 90\\ |\theta_{\mathrm{protoplast}}-\theta_{\mathrm{cellulose}}|-90 & if|\theta_{\mathrm{protoplast}}-\theta_{\mathrm{cellulose}}|>90 \end{array}\right.\right.$$

where θ_protoplast_ and θ_cellulose_ are the angles of the major axis of the protoplast fit to an ellipse and the mean angle of the skeletonized cellulose microfibril, respectively.

To estimate the variance of cellulose orientation, the parallelness of cellulose microfibrils was measured as previously described (Ueda et al., 2010). Parallelness (*P*) was defined as

$$P=\frac{|n_{0}-n_{90}\left|+\right|n_{45}-n_{135}|}{n_{0}+n_{45}+n_{90}+n_{135}}$$

where *n*_0_, *n*_45_, *n*_90_ and *n*_135_ are the number of pixel pairs that form 0, 45, 90, and 135° angles, respectively.

All measurements were performed using ImageJ plug‐ins: LpxLineFeature, which is invoked by Lpx_Filter2d plug‐ins (filter = lineFilters, linemode = lineFeature) in the LPixel ImageJ plugins package. *N*_cellulose_, $\overset{¯}{i}$, θ_cellulose_, *P*, and *S* were measured as i_nPix, i_mean, a_avgTheta, a_normAvgRad, i_stddevPerMean, and i_skewness by LpxLineFeature. θ_protoplast_ was measured from manually segmented protoplast images using ImageJ‐Analyze‐Measure.

## 3 RESULTS

### 3.1 Acquisition and quantification of image data for the cellulose network during cell wall regeneration

**Figure 1:**
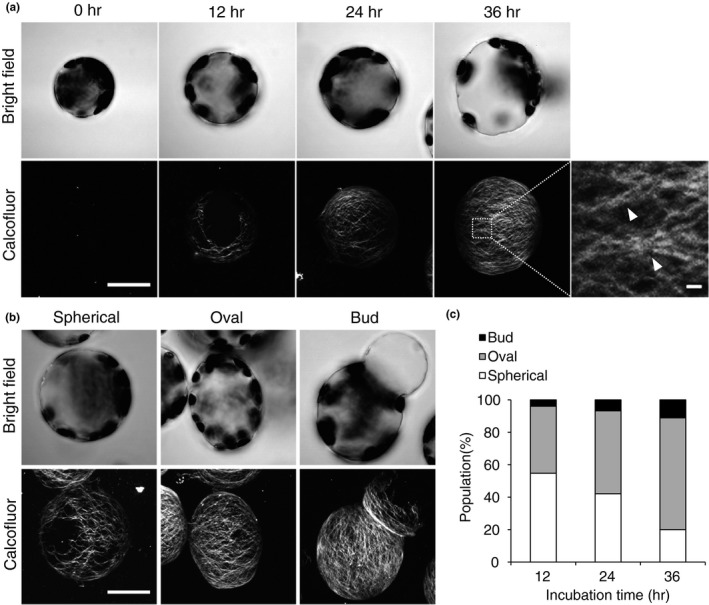
Cell wall regeneration in protoplasts derived from mesophyll cells of Arabidopsis rosette leaves. (a) Time course of cell wall regeneration in protoplasts. The protoplasts were incubated for 0, 12, 24, or 36 hr and stained with Calcofluor. Stained cells were observed under a bright‐field microscope (top) or a laser scanning confocal microscope (bottom). Inset in the image of protoplasts incubated for 36 hr is magnified on the right; arrowheads indicate bundles of cellulose fibrils. (b, c) Shapes of protoplasts observed under a bright‐field (top) or scanning confocal microscope (bottom) (b), and the population (%) of protoplasts of each shape (c). Bar = 20 μm or 1 μm (magnified image in (a)). *n* ≥ 126

Calcofluor/Cellufluor (Nagata and Takebe 1970; Hayashi et al.*,* 1986; Shea et al., 1989; Fisher and Cyr 1998) and Pontamine Fast Scarlet 4 BS (S4B) (Anderson et al., 2010; Peng, Zhang, Cheng, Fan, & Hao, 2013; Xiao, Zhang, Zheng, Cosgrove, & Anderson, 2016) are often used for fluorescent staining of cellulose. In this study, we chose Calcofluor because higher‐resolution fluorescent images of cellulose without autofluorescence signals are often acquired more readily using this dye compared with S4B (*cf*. Figure 1a, Fig. S1).

To confirm the specific binding of Calcofluor to nascent cellulose, we used this dye to stain protoplasts undergoing cell wall regeneration for 24 hr in the presence or absence of isoxaben, a potent inhibitor of cellulose synthesis (Tateno, Brabham, & DeBolt, 2016). Little or no fluorescence was detected in protoplasts incubated in the presence of isoxaben (Fig. S2), indicating that the fluorescence images represent the regenerated cellulose network.

During the 12‐ to 36‐h incubation period, the cellulose network spread over the entire surface of the protoplast, and individual threads in the network became intensively stained in a time‐dependent manner (Figure 1a). Each thread of the network consisted of bundles of thin fibrils (Figure 1a; arrowheads), indicating that the resolution of the confocal image is sufficient to visualize the fine structure of the cellulose network.

To ensure that callose signals were detected by the Calcofluor staining procedure, we stained protoplasts with aniline blue, which is a fluorescence dye specific for β‐1,3‐glucans, and compared the aniline blue‐staining pattern with the Calcofluor staining pattern. No fibril‐shaped fluorescent signals were observed in the cell wall‐regenerating protoplasts when they were stained with aniline, but dotlike signals were sometimes observed within 24 hr (Fig. S3, aniline blue). These dotlike signals were distinct from the fibril‐shaped signals and were also observed in Calcofluor‐stained protoplasts (Fig. S3, Calcofluor). This result indicates that callose, if any, is detected as a dot‐shaped signal, which can be readily distinguished from the fibrous signals of cellulose fibrils.

As the incubation proceeded, a portion of the protoplasts elongated and became either oval in shape (aspect ratio >1.05) or deformed and bud‐shaped (Figure 1b). The morphology of approximately 80% of the protoplasts changed during the 36‐h incubation period (Figure 1c). Given that the cell wall constrains cell shape in plants, this result indicates that the nascent cell wall had begun to determine cell shape at the beginning of the cell wall regeneration period, which was apparent in protoplasts at 12 hr of incubation under our conditions.

**Figure 2:**
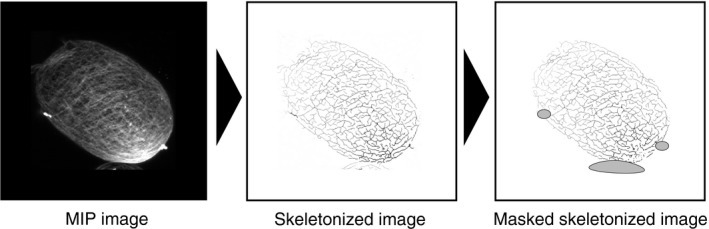
Workflow of image processing steps for obtaining the images used to derive the metrics to quantitatively evaluate cellulose network configuration

To quantitatively evaluate changes in the cellulose network configuration, we performed an image analysis technique that has successfully been used to quantify the configuration of the cytoskeleton (Higaki et al., 2010; Kimata et al., 2016; Ueda et al., 2010; Yoneda et al., 2007). Figure 2 shows the workflow of the image processing technique used in this analysis; the maximum intensity projection (MIP) images, which were obtained from serial optical sectional images, were skeletonized, and the skeletonized image was then masked to eliminate the signals derived from neighboring protoplasts and cell wall fragments, as well as the dot‐shaped signals of callose (Fig. S3). From the processed image data, the following metrics were measured: (i) total length of the skeletonized cellulose fibers (to evaluate the spread of the cellulose network), (ii) mean intensity in the skeletonized cellulose fibers (to evaluate cellulose levels), (iii) skewness of Calcofluor intensity distribution in the skeletonized cellulose fibers (to evaluate the extent of cellulose bundling), (iv) parallelness (to evaluate the variation in cellulose fiber orientation), and (v) average angle with respect to the long axis of the protoplast (to evaluate cellulose fiber orientation) (For a definition of individual metrics, see the Image processing section in Experimental Procedures.).

**Figure 3:**
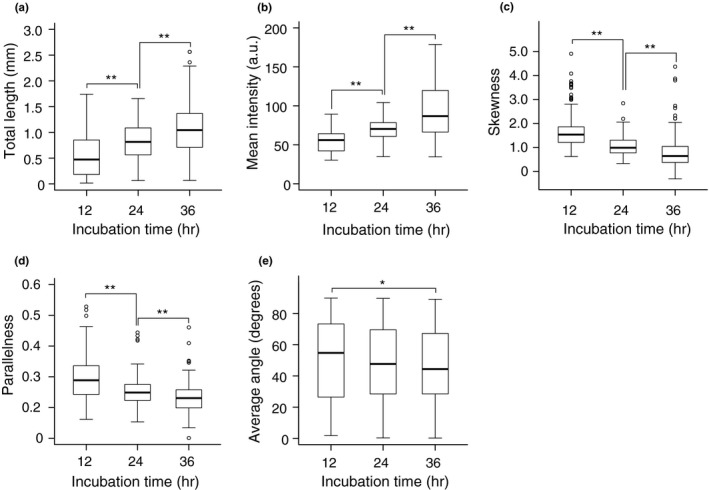
Time course of changes in cellulose configuration metrics during cell wall regeneration in protoplasts. (a–d) Protoplasts were incubated for 12, 24, or 36 hr, and total length (a), mean intensity (b), and skewness of intensity distribution (c) were measured from confocal optical images. a.u., arbitrary unit. (d, e) Parallelness (d) and average angle (e) of the cellulose network in oval‐shaped protoplasts incubated for 12, 24, or 36 hr. Significance was determined by Mann–Whitney test. **p < .01. *.01 ≤ *p* < .05. *n* ≥ 131

Total length, an indicator of the geometric spread of the cellulose network generated on the protoplast, increased during incubation (Figure 3a). Mean intensity, which reflects the strength of Calcofluor staining, also increased (Figure 3b). These results are consistent with our visual observations, as shown in Figure 1a.

Skewness of the intensity distribution, which is used as an indicator of cytoskeletal bundling (Higaki et al., 2010), decreased during incubation (Figure 3c). This might indicate a reduced bundling of microfibrils during the regeneration of the cellulose network.

To examine the relationship between protoplast elongation and cellulose orientation during protoplast culture, we measured the parallelness (which represents the variance of cellulose orientation) and average angle (which represents cellulose orientation) in oval‐shaped protoplasts. In these protoplasts, both metrics decreased during incubation, particularly the mean angle, which decreased to as low as 45 degrees (Figure 3d, e). These results indicate that the orientations of cellulosic fibers diverge from transverse to random orientation in protoplasts that elongate to become oval in shape. Parallelness or average angle of cellulose was plotted against the aspect ratio; however, these parameters showed little correlation with each other (Fig. S4), suggesting that the decrease in the parallelness or the mean angle of cellulose fibers is independent of protoplast elongation.

### 3.2 Verification of image analysis using agents that affect microtubule organization

To further verify the biological significance of these metrics, we investigated the effects of oryzalin and taxol, an inhibitor of microtubule polymerization and a microtubule‐stabilizing agent, respectively (Baskin, Wilson, Cork, & Williamson, 1994), on the cellulose network. To confirm the effects of these agents on microtubule organization, we used protoplasts derived from the Arabidopsis line *UBQ10::GFP‐TUB6*, which expresses tubulin labeled with GFP (Nakamura et al., 2004). We confirmed that in the absence of either agent, the *UBQ10::GFP‐TUB6*‐expressing protoplasts regenerated a cell wall and elongated during the 36‐h incubation period to the same extent as wild‐type protoplasts and that fluorescence images of GFP‐tubulin partially overlapped with the cellulose network visualized by Calcofluor (Fig. S5).

**Figure 4:**
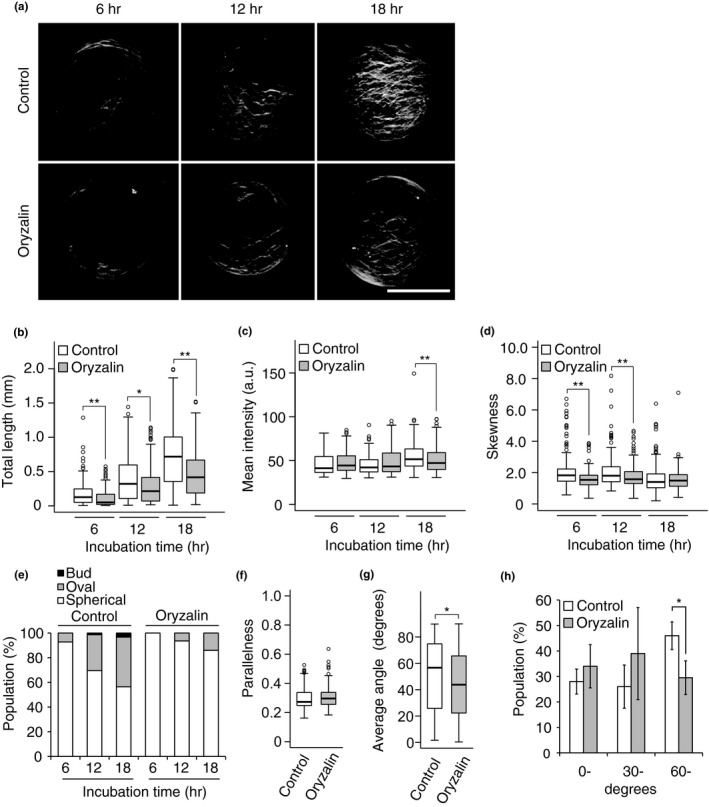
Effects of oryzalin on cell wall regeneration. (a) Representative images of the cellulose network in protoplasts incubated for 6, 12, or 18 hr in the presence or absence of oryzalin. After incubation, the protoplasts were stained with Calcofluor. (b–d) Total length (b), mean intensity (c), and skewness of intensity distribution (d) of the cellulose network regenerated from protoplasts incubated for 6, 12, or 18 hr in the presence or absence of oryzalin. (e) Changes in the populations of protoplasts with three different shapes during incubation for 6, 12, or 18 hr in the presence or absence of oryzalin. (f, g) Parallelness (f) and average angle (g) of the cellulose network with respect to the long axis of oval‐shaped protoplasts incubated for 24 hr in the presence or absence of oryzalin. (h) Effects of oryzalin on the population of oval‐shaped protoplasts with different average angles. Protoplasts were incubated for 24 hr, and oval‐shaped protoplasts were classified according to the average angle (0–29°, 30–59°, 60–90°). Individual populations are shown. Bar = 20 μm. Significance was determined by Mann–Whitney test. **p < .01, *.01 ≤ *p* < .05. *n* ≥ 106

Upon oryzalin application, GFP‐TUB6‐labeled cortical microtubules disappeared immediately, confirming that the polymerization of microtubules was inhibited by this agent (Fig. S6). Under these conditions, oryzalin inhibited the spread of the cellulose network during the 18‐h incubation period (Figure 4a). This inhibitory effect was confirmed by measuring the total length of skeletonized cellulose microfibrils (Figure 4b). On the other hand, mean intensity was only slightly affected by oryzalin treatment (Figure 4c). Skewness decreased at 6 and 12 hr of incubation in oryzalin (Figure 4d). The generation of oval‐shaped protoplasts was also inhibited by oryzalin treatment (Figure 4e). We also measured parallelness and average angle in oval‐shaped protoplasts after 24 hr of incubation. Whereas parallelness did not differ between oryzalin‐treated protoplasts and the control (Figure 4f), the average angle was significantly smaller in oryzalin‐treated protoplasts than in the control (Figure 4g). This reduced average angle is attributed to a reduced population of oval‐shaped protoplasts with higher average angles (60–90 degrees) in protoplasts incubated in the presence of oryzalin versus the control (Figure 4h).

**Figure 5:**
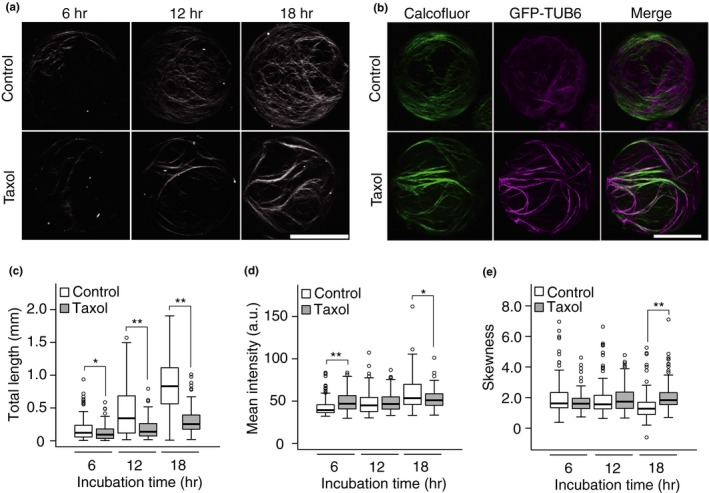
Effects of taxol treatment on cell wall regeneration. (a) Representative images of the cellulose network in protoplasts incubated for 6, 12, or 18 hr in the presence or absence of taxol. The protoplasts were stained with Calcofluor. (b) Cellulose network (Calcofluor) and cortical microtubules (GFP‐TUB6) in *UBQ10::GFP‐TUB6* protoplasts incubated for 12 hr in the presence (bottom) or absence (top) of taxol. (c–d) Total length (c), mean intensity (d), and skewness of intensity distribution (e) of the cellulose network regenerated from protoplasts incubated for 6, 12, or 18 hr in the presence or absence of taxol. Bar = 20 μm. Significance was determined by Mann–Whitney test. **p < .01, *.01 ≤ *p* < .05. *n* ≥ 104

When protoplasts were incubated with taxol, extensively bundled cortical microtubules appeared (Fig. S6) and bundled cellulose fibrils were generated during cell wall regeneration (Figure 5a). Analysis of the patterns of cellulose networks and cortical microtubules in *GFP‐TUB6* protoplasts using Calcofluor staining and GFP fluorescence clearly showed that bundled cortical microtubules predominantly colocalized with bundled cellulose fibrils (Figure 5b).

The increase in total length that occurs during cell wall regeneration (Figure 3a) was drastically reduced by taxol treatment to 70.5% at 6 hr, 40.8% at 12 hr, and 37% at 18 hr (Figure 5c), whereas the mean intensity increased at 6 hr, although it was only slightly different from that in the absence of taxol (Figure 5d). Skewness, which decreased during a prolonged (36 hr) incubation period (Figure 3c), was higher at 18 hr of taxol treatment than in the control (Figure 5e), suggesting that the skewness of the Calcofluor fluorescence intensity distribution is a useful parameter for the quantitative evaluation of bundling of cellulose fibers, as shown previously for cytoskeletons (Higaki et al., 2010).

## 4 DISCUSSION

### 4.1 Three features of our newly developed method

In this study, we developed an improved procedure for high‐resolution, quantitative imaging of the cellulose network regenerated from protoplasts. This new approach is characterized by three features:

First, a high rate (more than 80%) of cell wall regeneration can be achieved within 2 days using protoplasts derived from rosette leaves of Arabidopsis, and therefore, our technique can readily be used to assay the phenotypes of cell wall mutants as well as transformant lines of cell wall‐related genes.

Second, this method allows the observation of nascent cellulose networks using conventional laser scanning confocal microscopy, as the nascent cell wall regenerating on a protoplast is not optically obstructed by other preexisting cell wall components (Anderson et al., 2010; Peng et al., 2013; Xiao et al., 2016). Thus, the image resolution is sufficient to resolve the thin cellulose fibrils within each thread of the cellulose network at a resolution comparable to that obtained by atomic force microscopy imaging (Ding, Zhao, & Zeng, 2014; Zhang, Mahgsoudy‐Louyeh, Tittmann, & Cosgrove, 2014; Zhang et al., 2016, 2017).

Third, using this method, multiple features of the cellulose network can be measured from high‐resolution image data: (i) the geometric spread of the cellulose network, as represented by total length; (ii) the total amount of deposited cellulose, as represented by mean intensity; (iii) bundling of cellulose fibers, as represented by the skewness of intensity distribution; (iv) variation in cellulose fiber orientation, as represented by parallelness; and (v) the orientation of cellulose fibers against the major axis of the protoplast, as represented by average angle.

### 4.2 Total length as a measure of the spread of cellulose microfibril deposition

Total length and mean intensity increased during the 36‐h incubation (Figure 3a, b). The increase in total length of cellulose microfibrils was markedly reduced within 6 hr after the polymerization of cortical microtubules was either inhibited by oryzalin (Figure 4b) or stabilized by taxol (Figure 5c), whereas the mean intensity was not strongly affected by either agent. Given that cortical microtubules guide the insertion of the CSC into the plasma membrane, thereby directing the normal orientation of cellulose microfibrils (Crowell et al., 2009; Gutierrez, Lindeboom, Paredez, Emons, & Ehrhardt, 2009), it is reasonable to conclude that disrupting microtubule organization with oryzalin inhibited normal cellulose synthesis and failed to expand the cellulose network (Figure 4a). Stabilization of microtubules with taxol led to excess bundling of cellulose fibrils, resulting in bundled rather than spread‐out cellulose fibers (Figure 5a). Both situations were successfully detected by measuring total cellulose microfibril length (Figures 4b and 5c). Therefore, any loss‐of‐function or dominant‐negative mutation in factors required for the normal orientation of cellulose microfibrils, particularly those associated with the CSC, is expected to result in reduced total length compared with the control. Clearly, the total length of cellulose microfibrils is a good measure for the spread of cellulose microfibril deposition.

### 4.3 Skewness of intensity distribution as a measure of cellulose bundling status

Skewness, which represents an increase in the asymmetry of intensity distribution, has been used to evaluate the bundling of cytoskeleton (Higaki et al., 2010; Kimata et al., 2016). Our results indicate that the skewness of intensity decreases as cell wall regeneration proceeds (Figure 3c). This might reflect the reduction of bundling of cellulose microfibrils during the regeneration of the cellulose network (Figure 1a). In support of this notion, the decrease in the skewness metric was inhibited when microtubules were stabilized and bundled by taxol treatment (Figure 5e), which also inhibited the increase in the total length metric (Figure 5e). The negative correlation between the skewness of intensity distribution and the maturation of the cellulose network could be explained by a decrease in asymmetry as the number of cellulose microfibrils decreases during network maturation if a single cellulose fibril segment visualized by Calcofluor staining consists of several cellulose microfibrils.

Together, these results suggest that the extent of bundling of cellulose fibrils, which reflects the intensity distribution of the Calcofluor‐stained network pattern, can be represented by the skewness of intensity. Thus, we conclude that skewness is a useful measure for quantitatively evaluating cellulose bundling status. Thus, skewness could be used as a criterion when screening factors or enzymes affecting microtubule organization in the cytoplasm, as well as factors that affect interactions between cellulose microfibrils in the cell wall. These factors might include enzymes responsible for modifying cellulose–cellulose interactions, such as cellulose endotransglycosylase (Shinohara et al., 2017).

### 4.4 Quantification of cellulose orientation

The decrease in both the parallelness and the mean angle during the 12‐ to 36‐h incubation period indicates that cellulose fibrils become more randomly oriented as cell wall regeneration proceeds. Recently, Zhang et al. (2016) examined the nanoscale and mesoscale structures of the outer cell wall of onion scale epidermis using atomic force microscopy and showed that cellulose microfibrils are oriented in parallel within a lamella but vary by ~30 to 90° between adjacent lamellae, indicating that the wall has a crossed polylamellate structure. It is therefore plausible that the randomly oriented cellulose fibrils in the nascent wall surface of the protoplast may represent the process of formation of a crossed polylamellate structure.

The orientation of cellulose fibrils regenerated from protoplasts was measured in a previous study (Yoneda et al., 2007), but the long axis of the cell was not reflected in these measurements. In the current study, we determined the angles between cellulose fibers and the long axis in oval‐shaped protoplasts and defined the average angle, which declined when the cortical microtubule was disrupted by oryzalin (Figure 4g). This oryzalin‐induced decline in the average angle was accompanied by the inhibited elongation of protoplasts (Figure 4e). These results are consistent with the widely accepted concept that plant cells expand or elongate in a direction perpendicular to the orientation of cellulose microfibrils, which are deposited in the same orientation as cortical microtubules (Baskin, 2005; Paredez et al., 2006; Somerville et al., 2004). Thus, average angle metrics can be used as an indicator of cell elongation.

## 5 CONCLUSIONS

Three metrics, that is, total length, skewness of intensity distribution, and average angle of cellulose fibers, which can be measured easily from confocal imaging data acquired during the early stage of cell wall regeneration in protoplasts, can be used as quantitative criteria for the organization status of nascent cellulose microfibrils. As this procedure is based on the use of protoplasts derived from mesophyll cells of Arabidopsis rosette leaves, it can easily be adapted to screen for genes or agents that might affect cellulose organization during cell wall construction. The use of a reverse genetics approach based on our newly developed quantitative imaging technique using omics data for cell wall formation in protoplasts (Chupeau et al., 2013; Kwon, Yokoyama, & Nishitani, 2005) will provide a novel opportunity to identify key factors that contribute to the construction and/or regulation of the cellulose network (Yokoyama, Kuki, Kuroha, & Nishitani, 2016). Taken together, our new quantitative imaging approach, combined with the use of Arabidopsis protoplasts, is applicable to a broad range of studies on cell wall formation, particularly for screening genes responsible for cell wall construction and its regulation.

## AUTHOR CONTRIBUTIONS

H.K. performed the experiments and data acquisition with occasional discussions with T.H., R.Y., T.K., N.S., and S.H. under supervision of K.N.; H.K. and T.H. conducted data analysis; H.K., T.H., and K.N. put together the manuscript with discussions with R.Y., T.K., N.S., and S.H.
