# Supplementary material for: Quantitative confocal imaging method for analyzing cellulose dynamics during cell wall regeneration in Arabidopsis mesophyll protoplasts
Source: Plant Direct. 2017 Dec 27;1(6):e00021. doi: 10.1002/pld3.21 (PMC6508514; doi:10.1002/pld3.21)
Supplement: Supplementary file 1 [file PLD3-1-e00021-s001.pdf]

Supplementary Figures for

Quantitative confocal imaging method for analyzing cellulose dynamics during cell wall regeneration in Arabidopsis mesophyll protoplasts

by

Hiroaki Kuki, Takumi Higaki, Ryusuke Yokoyama, Takeshi Kuroha, Naoki Shinohara, Seiichiro Hasezawa, and Kazuhiko Nishitani

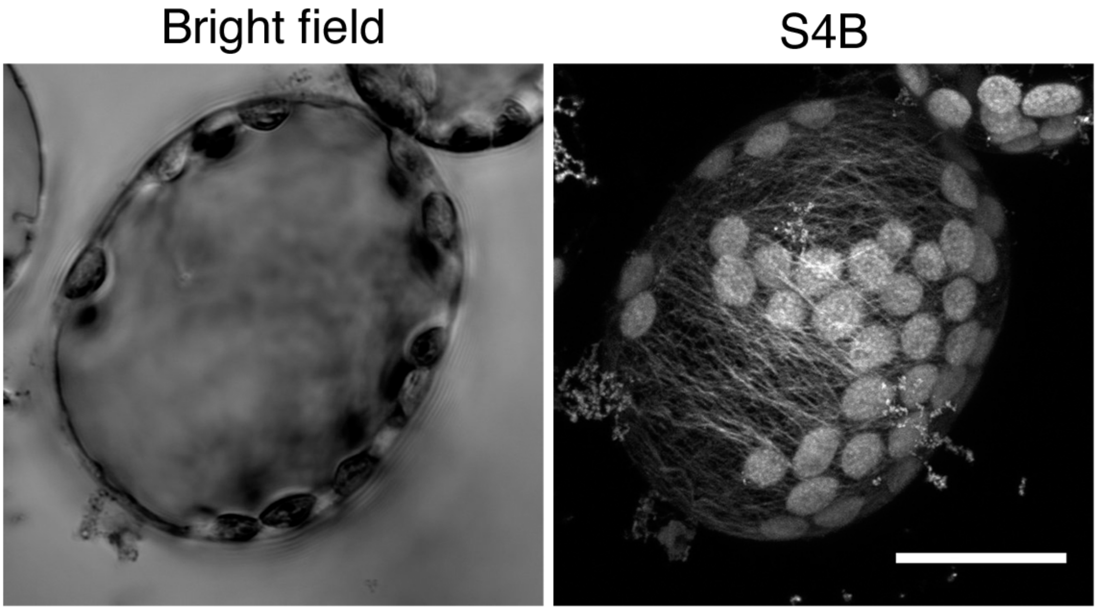

Figure S1. S4B staining of nascent cell wall regenerated from protoplasts. Protoplasts were incubated for 24 h and stained with 0.03% S4B. (left) bright field image; (right) the maximum intensity projection image ranging from the top to the middle of the protoplast with 0.5 μm increment. Scal bar = 20 μm.

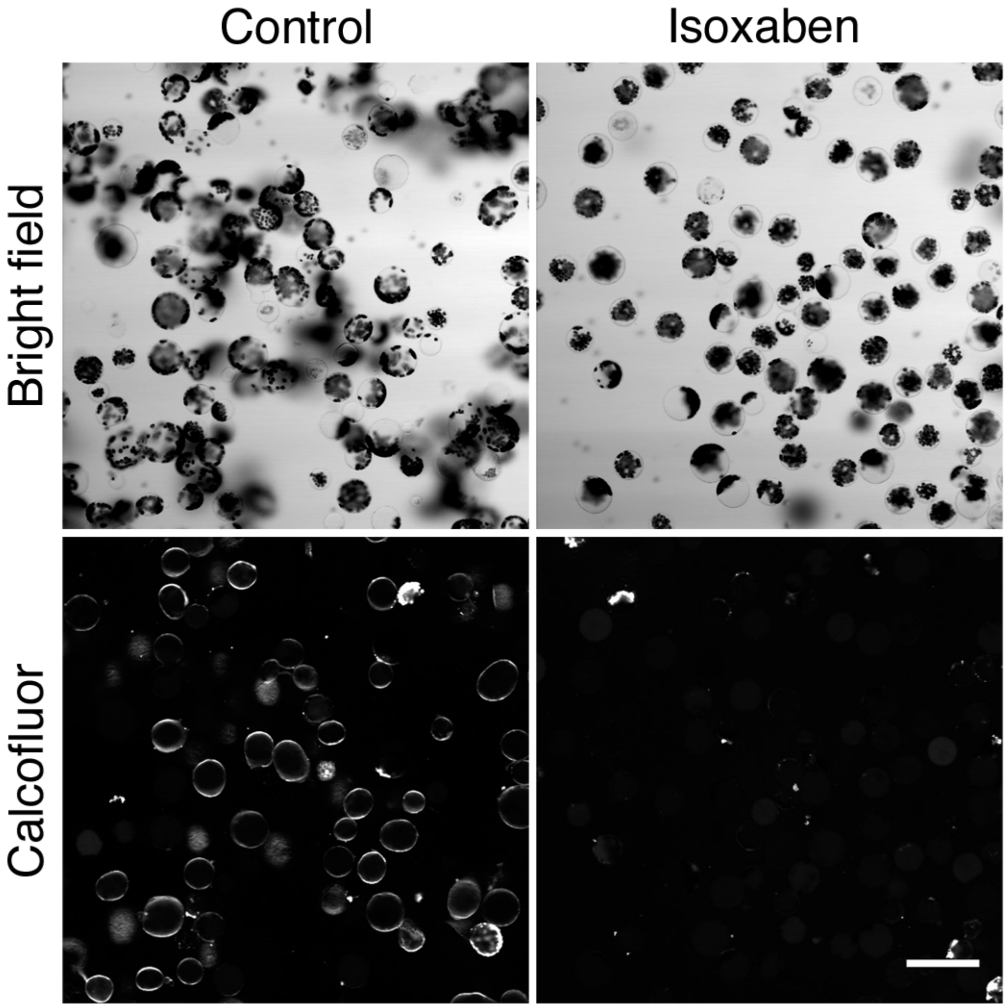

Figure S2. The effects of isoxaben treatment on cell wall regeneration.
Protoplasts were incubated in the absence (left) or presence (right) of isoxaben for 24 h, followed by staining with calcofluor. Bright field images (top) and confocal images of calcofluor fluorescence (bottom), are shown. Scale bar = 100 μm.

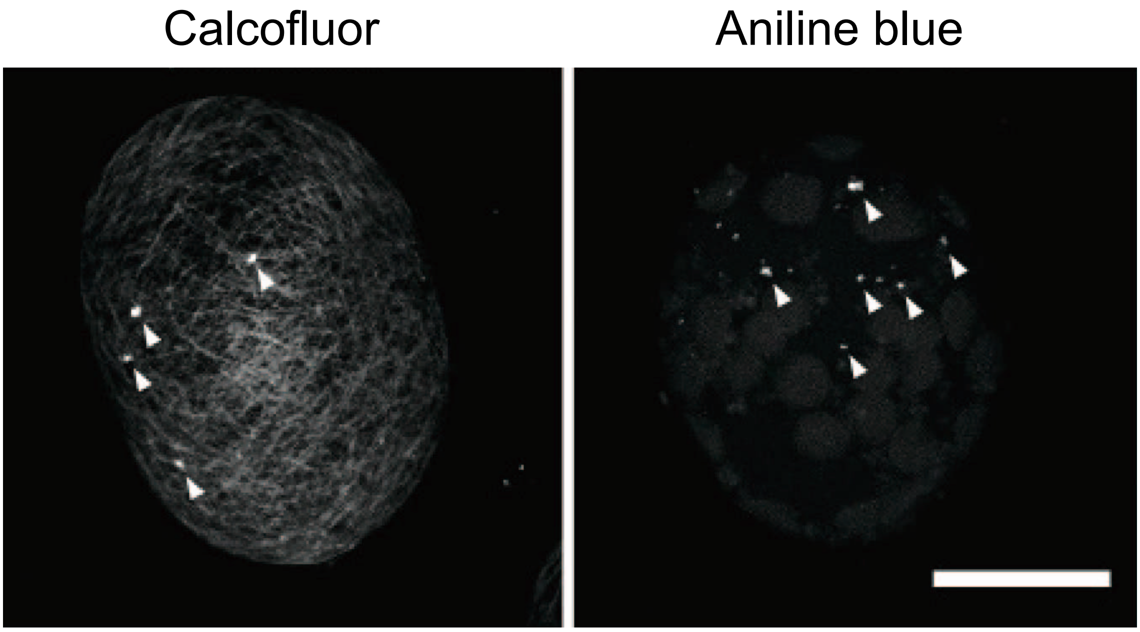

Figure S3. Comparison of the calcofluor and aniline staining patterns to distinguish cellulose from callose.

After incubation for 24 h, protoplasts were stained with either calcofluor (left) or aniline blue (right), followed by observation under a fluorescence microscope. The dot-like fluorescent signals (indicated by Arrowheads) were observed by calcofluor or aniline blue staining, indicating that they are callose signals. Scale bar = 20 μm.

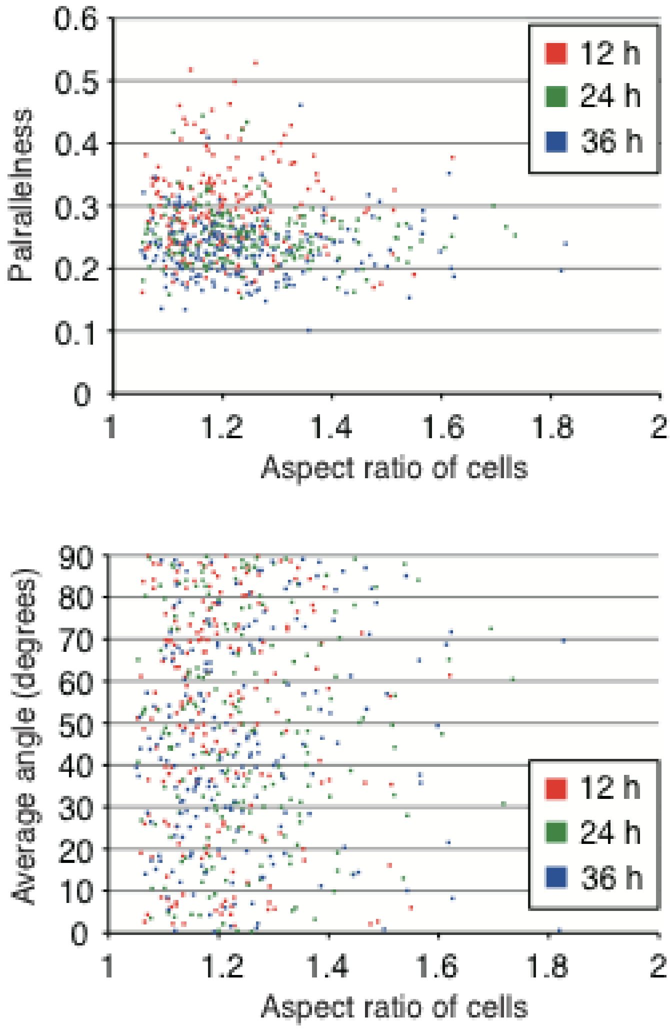

Figure S4. Correlation between parallelness or average angle of cellulose fibrils and aspect ratio of protoplasts.

Protoplasts were incubated for 12 (red dots), 24(green dots), or 36 h (blur dots), and scatter plot of parallelness (top) or average angle (bottom) versus aspect ratio of protoplats were drawn. n = 200.

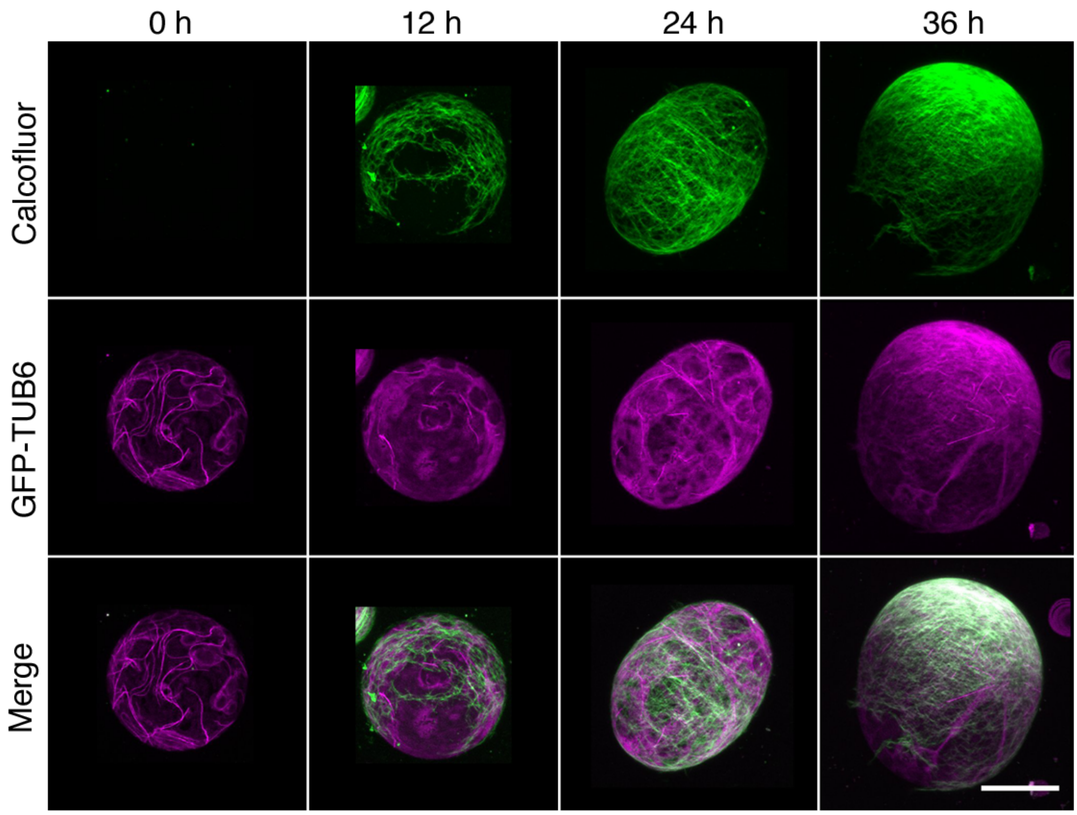

Figure S5. Time course of cell wall regeneration and cortical microtubules organization in *UBQ10 :: GFP-TUB6* expressing protoplasts in the absence of oryzalin or taxol.

UBQ10 :: GFP-TUB6 expressing protoplasts were incubated for 0, 12, 24 or 36 h (from left to right) and stained with calcofluor. Fluorescent images of calcofluor (top) and GFP-tubulin (middle) were acquired, and merged images (bottom) were created. Sale bar = 20 μm.

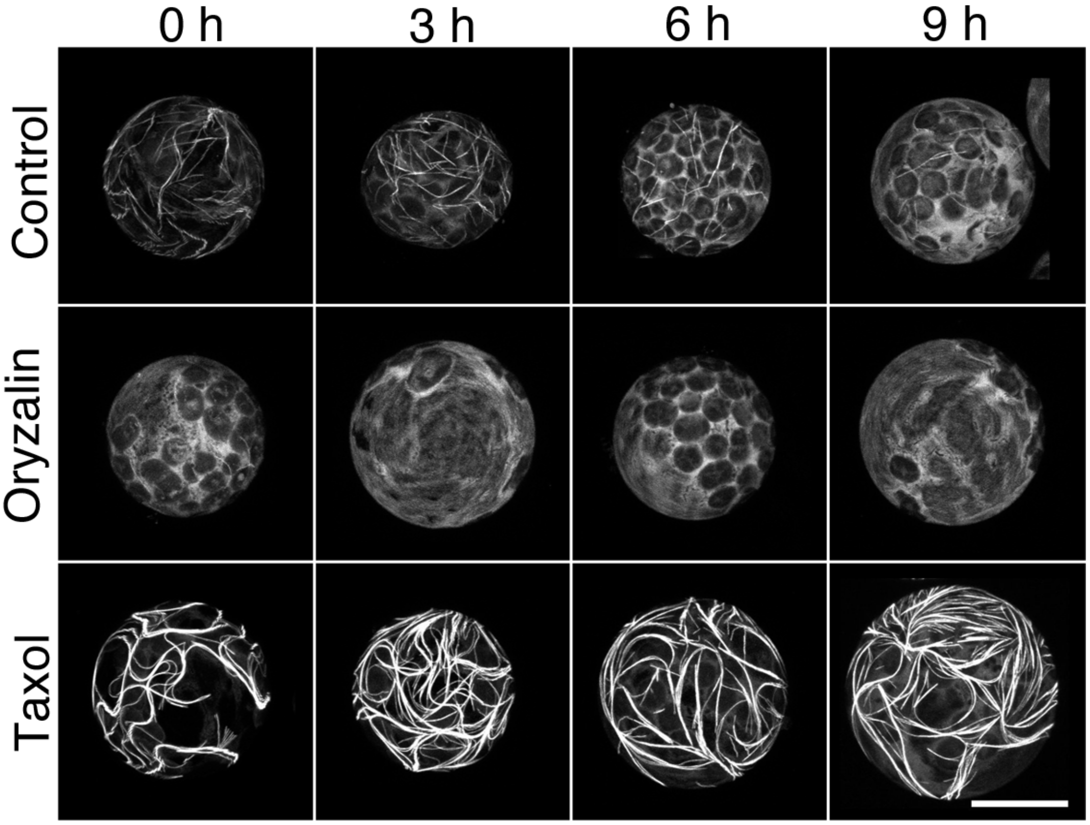

Figure S6. Cortical microtubules in the regenerating cell walls of protoplasts. *UBQ10::GFP-TUB6* expressing protoplasts were incubated for 0, 3, 6, or 9 h (from left to right) in the presence of oryzalin (middle) or taxol (bottom) or in the absence of either agent (top). Scale bar = 20 µm.
